# Application of Mixed Reality for Ophthalmic Clinical Skills and Diagnosis: Prospective Study

**DOI:** 10.2196/71338

**Published:** 2026-03-03

**Authors:** Chun Jin Marcus Tan, Wei Wei Dayna Yong, Hui'En Hazel Anne Lin, Jaslyn Oh, How Sheng Rubin Yong, Fang Mei Jayme Khew, Liang Shen, Yujia Gao, Wei Chieh Alfred Kow, Yih Chung Tham, Dianbo Liu, Ching-Yu Cheng, Kee Yuan Ngiam, Yew Sen Yuen, Ray Manotosh, Eng Tat Khoo, Teck Chang Victor Koh, Woon Teck Clement Tan

**Affiliations:** 1 Department of Ophthalmology, National University Hospital, 5 Lower Kent Ridge Road, Singapore, 119074, Singapore, 65 6908 2222; 2 Centre for Innovation and Precision Eye Health, Yong Loo Lin School of Medicine, National University of Singapore, Singapore, Singapore; 3 Biostatistics Unit, Yong Loo Lin School of Medicine, National University of Singapore, Singapore, Singapore; 4 Division of Hepatobiliary & Pancreatic Surgery, National University Hospital, Singapore, Singapore; 5 Division of General Surgery (Thyroid & Endocrine Surgery), National University Hospital, Singapore, Singapore; 6 Engineering Design and Innovation Centre, National University of Singapore, Singapore, Singapore

**Keywords:** mixed reality, medical education, HoloLens, ocular motility, ophthalmology teaching

## Abstract

**Background:**

Mixed reality has the potential to transform delivery of medical education. With tools such as HoloLens 2, educators can create immersive, interactive simulations that enable students to practice and engage with real-world scenarios in a controlled environment.

**Objective:**

We postulated that a hybrid ophthalmology curriculum incorporating EyelearnMR (a simulation application) would be noninferior to traditional teaching. We compared learning outcomes and obtained user feedback.

**Methods:**

This was a single-blind, cluster-randomized prospective study. Fourth-year medical students were organized into batches and then assigned to 2 groups: EyelearnMR and control arms. We used a quasi-randomized design with alternation allocation based on clinical grouping. The intervention group had an additional 2 hours of practice with the EyelearnMR devices. During the second week of their posting, a video assessment (5 scenarios with 17 multiple-choice questions) was conducted for both groups—mid-posting for the intervention group and at the end of the posting for the control group. The rationale for assessing the intervention group earlier, in addition to setting a higher bar for EyelearnMR, was to allow for the provision of outcomes showing noninferiority between both groups. In the event of noninferiority, we could demonstrate that EyelearnMR can replace some degree of traditional clinical teaching, even with a shorter total clinical exposure time. Students in the control group were allowed to experience the Eyelearn MR modules for 2 hours at the end of the posting. Both groups were asked to complete the User Experience Questionnaire.

**Results:**

This study was funded in February 2023, and recruitment took place from July 2023 to January 2024. A total of 54 students were recruited—24 (44.4%) in the control arm and 30 (55.6%) in the EyelearnMR arm. The EyelearnMR group performed significantly better than the control group (median scores of 16, IQR 15-17, and 15, IQR 14-15, respectively; *P*=.03; Mann-Whitney *U* test). A total of 100% (30/30) of the students in the EyelearnMR group scored full marks (3/3) for the technique portion, compared to 70.8% (17/24) of the students in the control group (*P*=.002). There was no statistically significant difference between the groups for the examination (*P*=.13) and pathology (*P*=.33) portions. This was despite the EyelearnMR group having a reduced clinical time of 7 days compared to 10 days in the control group. The User Experience Questionnaire showed positive evaluations for attractiveness (mean 1.413, SD 0.969), efficiency (mean 0.822, SD 1.068), dependability (mean 1.087, SD 0.801), stimulation (mean 1.577, SD 0.845), and novelty (mean 1.606, SD 0.967).

**Conclusions:**

EyelearnMR with traditional teaching was noninferior to traditional teaching alone. It provided a comparable experience and supported learning objectives equally. It is an effective supplementary teaching tool in ophthalmic education and may confer additional learning benefits beyond a traditional clinical posting.

## Introduction

Mixed reality (MR) is a new concept that merges the real and virtual worlds [1,2], having the potential to transform the delivery of medical education [3]. This technology could address challenges faced in the delivery of high-quality medical education, including accessibility, consistency, quality, and cost [4,5]. The Microsoft HoloLens 2 is a commercially available MR headset that allows for bidirectional communication with multiple remote users via video, voice, and MR composites [6]. This technology has been used previously in various clinical and educational scenarios, including anatomical teaching, perioperative planning, and surgical training [4,5,7-16]. This technology has also been used to augment and supplement medical student clinical skill teaching and examination [17-19], allowing for the creation of immersive multisensory (audio, visual, and tactile) content to replicate real-world scenarios. MR allowed students to practice techniques and refine their skills in a controlled environment, leading to fewer errors and increased confidence in real-world scenarios.

The traditional methods of teaching students how to perform an eye movement examination and detect abnormalities of ocular motility include clinical demonstrations, slide and video presentations, and case-based teaching in the wards and clinics. The last scenario, while the most realistic, is also opportunistic and inconsistent.

**Figure 1.**
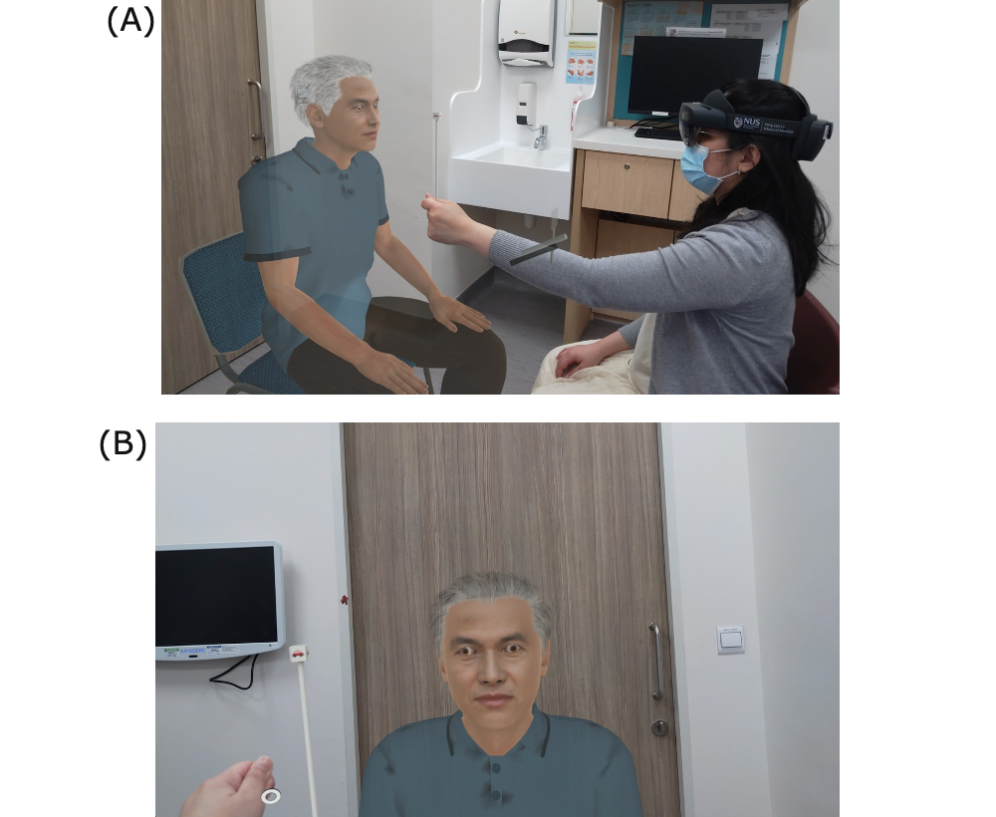
Screen capture of (A) the instructor’s view from EyelearnMR and (B) the user’s view from EyelearnMR.

EyelearnMR is an MR simulation application designed to teach and assess ocular examination via a wearable headset. The application consists of a hologram 3D patient, a Lang fixation cube for ocular motility examination, and an animation logic that models eye movement when the patient’s eyes are fixating on the cube. It is deployed on the Microsoft HoloLens 2 device and offers a 3D superimposition of simulated patients in a physical space (Figure 1). MR simulation enables technology-enhanced learning, which provides opportunities for trainees to acquire, develop, and maintain knowledge, skills, and behavior via experiential learning. MR simulation in medical education has several advantages, including precise tracking of quantifiable performance parameters in a clinical examination and consistent reproduction of clinical signs without fatiguing a volunteer patient. It can also be readily deployable in large class sizes outside of a traditional classroom with fewer physical constraints.

A review by Cook et al [20] showed that simulation-based learning can achieve comparable outcomes to those of traditional methods in other fields of medical education. As such, we came up with a study that allowed us to evaluate the feasibility and reception of an MR-based ophthalmology curriculum in honing ophthalmic skills, providing evidence to support the wider adoption of MR in medical curricula. Moreover, the introduction of the MR-based ophthalmology curriculum could help address challenges in ophthalmology training posed by an increasing number of students, limited faculty tutors, and reduced patient exposure.

In this study, we aimed to compare students’ performance on a bespoke video-based assessment between a curriculum incorporating EyelearnMR and a traditional clinical teaching–only curriculum. The assessment evaluated 3 key dimensions of ocular motility learning: examination technique, recognition of clinical signs, and diagnostic reasoning. We postulated that a hybrid ophthalmology curriculum incorporating EyelearnMR would be noninferior to traditional teaching. In addition, we evaluated student perceptions of the MR platform through the validated User Experience Questionnaire (UEQ).

## Methods

### Overview

This was a single-blind, cluster randomized prospective study. We included undergraduate medical students (fourth year and 10 clinical groups in total; age range 23 to 26 years) posted to the Department of Ophthalmology, National University Hospital, Singapore, for their 2-week ophthalmology rotation. The clinical groups (each group comprised 6-7 students) were randomized into 2 arms (5 clinical groups randomized into the EyelearnMR arm and the other 5 clinical groups randomized into the control arm). We used the quasi-randomized design using alternation allocation. For feasibility, clinical groups were randomized, and all students in the same clinical group who agreed to participate in the study were in the same arm. The EyelearnMR learning module consists of 6 different scenarios of ocular motility simulated in a model patient, which the students were free to peruse within the practice session of 2 hours. The module was presented to the students in a structured and guided fashion in accordance with pedagogical best practices.

Both groups underwent the usual 2-week ophthalmology rotation with 6 preset tutorials for various topics and 9 days of clinical exposure. The intervention group had an additional 2 hours of practice with the EyelearnMR devices. Six HoloLens units were available for the study. During the second week of their posting, a 30-minute assessment (5 videos were played, and students had to answer 17 multiple-choice questions related to ocular motility in the domains of examination technique, pathology, and examination signs) was conducted for both the control and intervention groups. To our knowledge, no standardized assessments using psychometric data and benchmarks are available. As such, a bespoke assessment was created. We used “remember,” “understand,” and “apply” from Bloom’s taxonomy hierarchy to come up with the questions for the scenarios. The clinical assessment was intentionally designed to focus on core competencies relevant to ocular motility—specifically, examination technique, recognition of clinical signs, and diagnostic reasoning. A total of 17 structured multiple-choice questions were administered across 5 video-based clinical scenarios, each designed to assess different facets of performance. The questions were vetted by 2 senior neuro-ophthalmologists in clinical practice, ensuring clinical validity and educational relevance. The following is a breakdown of the scenarios:

- Scenario 1: normal patient to test ocular motility assessment technique
- Scenario 2: patient with left partial cranial nerve 3 palsy
- Scenario 3: patient with right cranial nerve 6 palsy
- Scenario 4: patient with thyroid eye disease (lid retraction and ophthalmoplegia)
- Scenario 5: patient with left internuclear ophthalmoplegia

**Figure 2.**
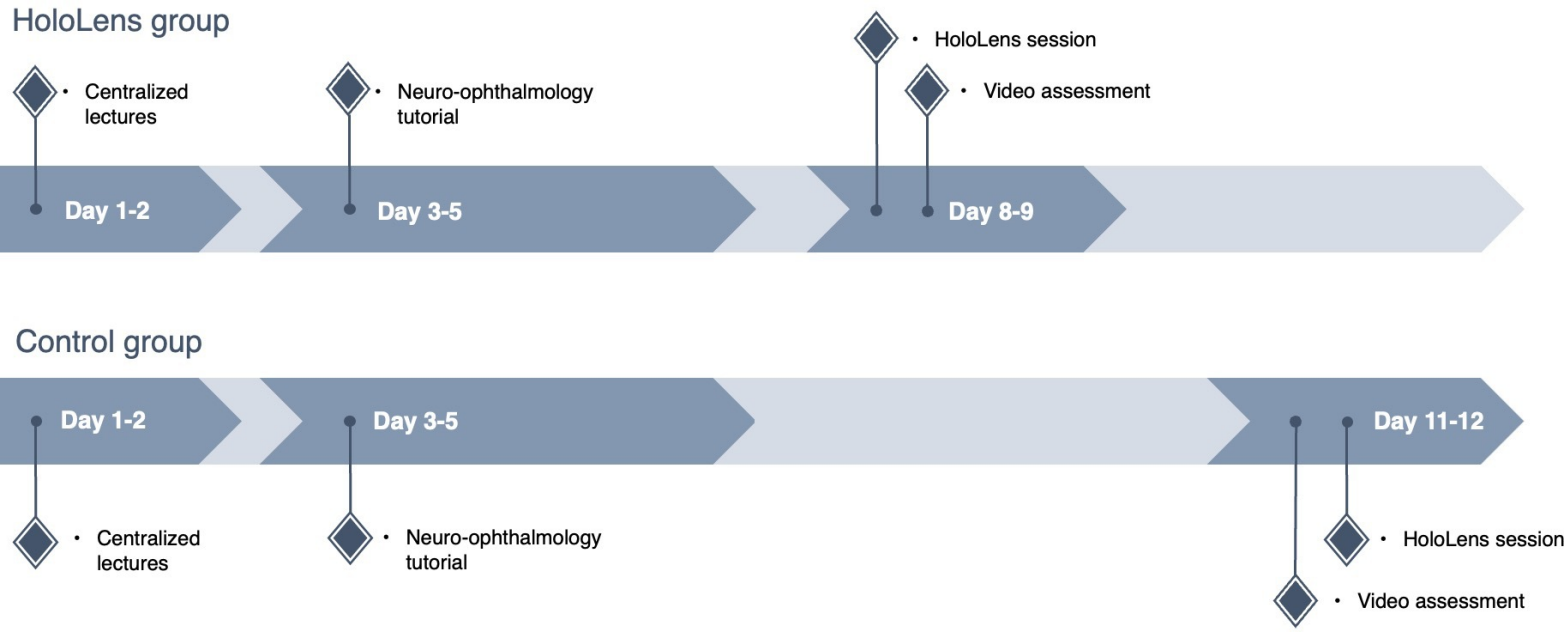
User study timeline.

The assessment was conducted mid-posting for the intervention group and at the end of the posting for the control group. The assessment questions were not shared with the groups beforehand. The rationale for assessing the intervention group earlier, in addition to setting a higher bar for EyelearnMR, was to allow for the provision of outcomes showing noninferiority between both groups. In the event of noninferiority, we could demonstrate that EyelearnMR is able to replace some degree of traditional clinical teaching even with a shorter total clinical exposure time. For the EyelearnMR group, the 2-hour EyelearnMR session and the 30-minute video-based assessment were scheduled on days 8 and 9 of the 2-week posting (Figure 2). For the control group, the same assessment was conducted on days 11 and 12, coinciding with the usual end-of-posting evaluation. This schedule reflected how EyelearnMR was intended to be implemented in practice: as a mid-rotation consolidation session that could be integrated into the existing timetable without displacing core teaching activities or overloading the final days of the posting.

Students in both groups were asked to fill out the UEQ [21]. Students in the control group were given the opportunity to experience the EyelearnMR modules for 2 hours at the end of the posting, following which they were also asked to fill out the UEQ.

A sample of the assessment questions and the UEQ can be found in Multimedia Appendix 1.

### Statistical Analysis

Data were analyzed descriptively first. Means and SDs or medians and ranges were reported for the numerical variables, whereas frequencies and percentages were reported for the categorical or ordinal variables. Comparison of scores between groups was analyzed using the Mann-Whitney *U* test. The Fisher exact test was used to compare the test results on technique, examination, and pathology-related knowledge. *P* values of <.05 were considered statistically significant. Results from the UEQ were evaluated using the data analysis tools provided by the UEQ team [22]. The range of the scales is between −3 (“horribly bad”) and +3 (“extremely good”). Values between −0.8 and 0.8 represent a neutral evaluation of the corresponding scale, values of >0.8 represent a positive evaluation, and values of <−0.8 represent a negative evaluation [21]. Statistical analysis was carried out using SPSS (version 29.0.0; IBM Corp).

### Ethical Considerations

This study was approved by the National University of Singapore Learning and Analytics Committee on Ethics (L2022-09-02). Informed consent was obtained and participants’ confidentiality was maintained by not having any personal data recorded.

## Results

Recruitment was conducted from July 2023 to January 2024 and was completed as of submission of this manuscript.

A total of 54 fourth-year medical students (age range 23-26 years) were recruited for this study: 24 (44.4%) in the control arm and 30 (55.6%) in the EyelearnMR arm. The median assessment score was 15 (IQR 14-15) for the control group and 16 (IQR 15-17) for the EyelearnMR group (*P*=.03). The assessment questionnaire consisted of items about testing techniques, examination, and pathology-related knowledge (as shown in Multimedia Appendix 1). As shown in Table 1, a total of 100% (30/30) of the students scored full marks (3/3) for the technique portion in the EyelearnMR group compared to 70.8% (17/24) of the students in the control group, and this was statistically significant (*P*=.002). There was no statistically significant difference between the groups for the examination and pathology portions.

**Table 1.** Fisher exact test results according to subgroup analysis.

Question	Control group (n=24), n (%)	MR^a^ group (n=30), n (%)	Fisher exact test *P* value
Technique (out of 3 points)			.002
2 points	7 (29.2)	0 (0.0)	
3 points	17 (70.8)	30 (100.0)	
Examination (out of 8 points)			.13
4 points	1 (4.2)	1 (3.3)	
5 points	3 (12.5)	3 (10.0)	
6 points	9 (37.5)	9 (30.0)	
7 points	9 (37.5)	11 (36.7)	
8 points	2 (8.3)	11 (36.7)	
Pathology (out of 6 points)			.33
4 points	1 (4.2)	1 (3.3)	
5 points	7 (29.2)	4 (13.3)	
6 points	16 (66.7)	25 (83.3)	

a MR: mixed reality.

**Figure 3.**
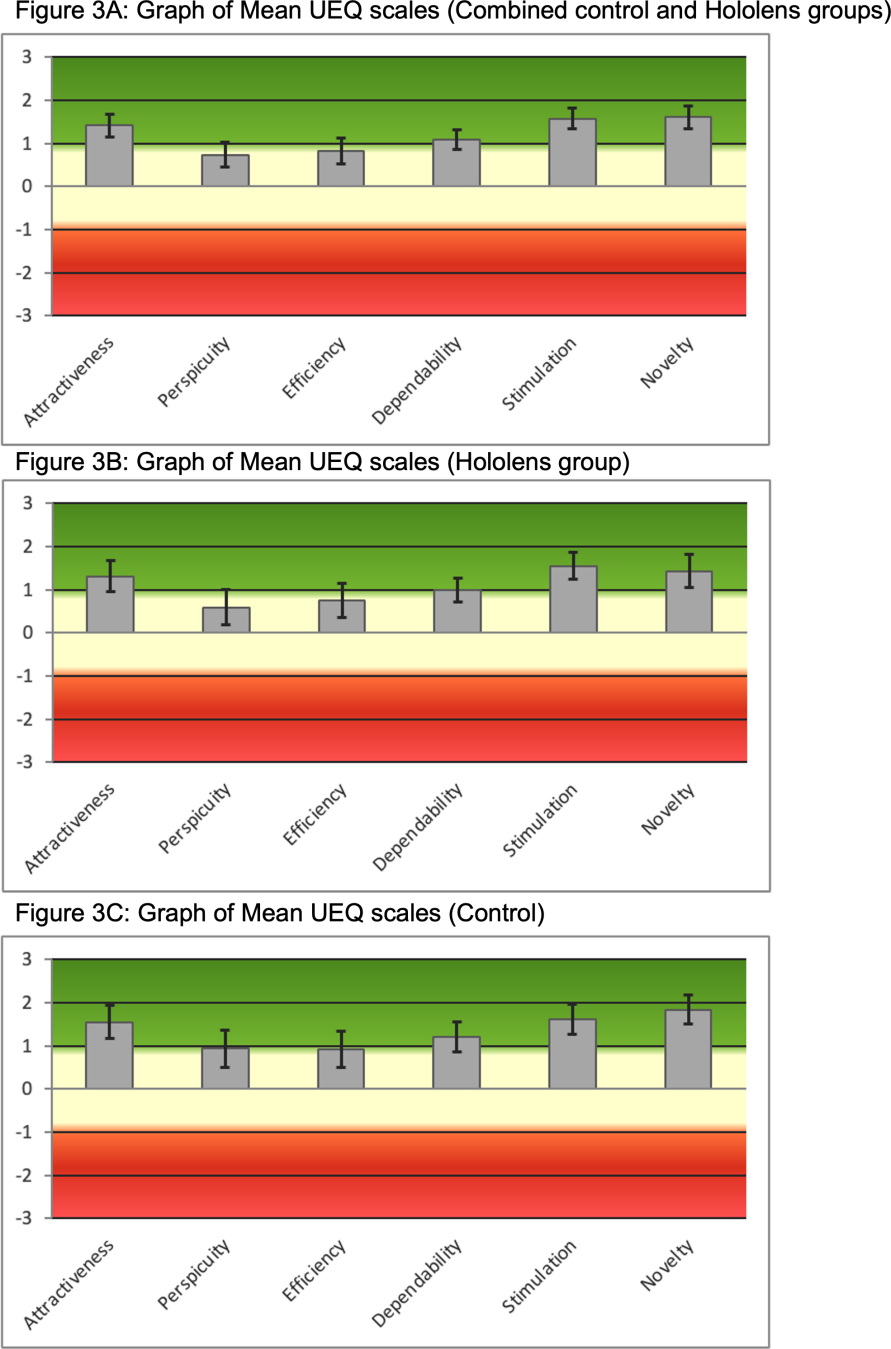
Graphs of mean User Experience Questionnaire scales: (A) combined control and EyelearnMR groups; (B) EyelearnMR group; and (C) control group.

The results from the UEQ are shown in Tables2  3, as well as in Figure 3. Regarding the combined evaluation, attractiveness, efficiency, dependability, stimulation, and novelty received a positive evaluation. Perspicuity received a neutral evaluation. Statistical analysis using a simple 2-tailed *t* test showed no statistically significant difference between the HoloLens and control groups for attractiveness, perspicuity, efficiency, dependability, stimulation, and novelty (*P*=.37, *P*=.26, *P*=.57, *P*=.38, *P*=.79, and *P*=.12, respectively).

**Table 2.** Mean scores and variances of the User Experience Questionnaire items.

Item	Score (–3 to +3), mean (SD)	Variance	Lower bound	Upper bound	Scale
1^a^	1.0 (1.6)	2.6	“Annoying”	“Enjoyable”	Attractiveness
2^a^	1.6 (1.0)	0.9	“Not understandable”	“Understandable”	Perspicuity
3^a^	1.6 (1.6)	2.6	“Creative”	“Dull”	Novelty
4^b^	0.5 (1.6)	2.4	“Easy to learn”	“Difficult to learn”	Perspicuity
5^a^	1.5 (1.2)	1.4	“Valuable”	“Inferior”	Stimulation
6^a^	1.7 (1.0)	1.0	“Boring”	“Exciting”	Stimulation
7^a^	1.9 (1.0)	1.0	“Not interesting”	“Interesting”	Stimulation
8^b^	0.2 (1.4)	2.0	“Unpredictable”	“Predictable”	Dependability
9^b^	0.0 (1.5)	2.3	“Fast”	“Slow”	Efficiency
10^a^	2.0 (1.0)	1.1	“Inventive”	“Conventional”	Novelty
11^a^	1.6 (1.1)	1.1	“Obstructive”	“Supportive”	Dependability
12^a^	1.8 (1.0)	1.0	“Good”	“Bad”	Attractiveness
13^b^	0.0 (1.4)	1.9	“Complicated”	“Easy”	Perspicuity
14^a^	1.3 (1.1)	1.3	“Unlikable”	“Pleasing”	Attractiveness
15^a^	1.3 (1.2)	1.5	“Usual”	“Leading edge”	Novelty
16^a^	1.2 (1.2)	1.4	“Unpleasant”	“Pleasant”	Attractiveness
17^a^	1.0 (1.2)	1.5	“Secure”	“Not secure”	Dependability
18^a^	1.2 (1.2)	1.4	“Motivating”	“Demotivating”	Stimulation
19^a^	1.5 (1.1)	1.2	“Meets expectations”	“Does not meet expectations”	Dependability
20^b^	0.7 (1.5)	2.2	“Inefficient”	“Efficient”	Efficiency
21^a^	0.8 (1.6)	2.6	“Clear”	“Confusing”	Perspicuity
22^a^	1.1 (1.3)	1.7	“Impractical”	“Practical”	Efficiency
23^a^	1.5 (1.1)	1.2	“Organized”	“Cluttered”	Efficiency
24^a^	1.6 (1.0)	1.1	“Attractive”	“Unattractive”	Attractiveness
25^a^	1.6 (1.0)	0.9	“Friendly”	“Unfriendly”	Attractiveness
26^a^	1.5 (1.4)	1.9	“Conservative”	“Innovative”	Novelty

a Positive evaluation.

b Neutral evaluation.

**Table 3.** Mean scores and variances of the User Experience Questionnaire (UEQ) scales overall, for the HoloLens group, and for the control group.

UEQ scale	Score (–3 to +3), mean (SD)	Variance	Cronbach α coefficient
Overall			
Attractiveness^a^	1.413 (0.969)	0.94	0.92
Perspicuity^b^	0.736 (1.094)	1.2	0.8
Efficiency^a^	0.822 (1.068)	1.14	0.8
Dependability^a^	1.087 (0.801)	0.64	0.61
Stimulation^a^	1.577 (0.845)	0.71	0.78
Novelty^a^	1.606 (0.967)	0.93	0.72
sEyelearnMR group			
Attractiveness^a^	1.311 (1.01)	1.02	0.92
Perspicuity^b^	0.592 (1.15)	1.31	0.82
Efficiency^b^	0.75 (1.13)	1.28	0.86
Dependability^a^	1 (0.77)	0.6	0.6
Stimulation^a^	1.55 (0.86)	0.74	0.76
Novelty^a^	1.433 (1.04)	1.09	0.72
Control group			
Attractiveness^a^	1.553 (0.915)	0.84	0.93
Perspicuity^a^	0.932 (1.012)	1.02	0.76
Efficiency^a^	0.92 (0.992)	0.98	0.71
Dependability^a^	1.205 (0.84)	0.71	0.61
Stimulation^a^	1.614 (0.841)	0.71	0.83
Novelty^a^	1.841 (0.818)	0.67	0.71

a Positive evaluation.

b Neutral evaluation.

## Discussion

### Principal Findings

In this noninferiority study, a curriculum incorporating EyelearnMR performed at least as well as the traditional clinical teaching–only curriculum and overall supported comparable learning objectives. Our study demonstrates that EyelearnMR on the HoloLens 2 is an effective teaching tool for ocular motility examination. Students who used EyelearnMR achieved significantly higher scores on the video-based assessment compared with those who underwent traditional clinical teaching alone (median score 16 vs 15 out of 17; *P*=.03). Although the difference was statistically significant, the absolute gap of 1 point on a 17-point scale is small, and its practical or clinical relevance should be interpreted in context. There was a particularly marked difference in examination technique (*P*=.002). This was despite the EyelearnMR group having reduced clinical exposure time at the time of testing. The improved performance in the EyelearnMR group likely reflects the combined impact of structured deliberate practice, standardized exposure to key motility disorders, and consistent reproduction of clinical signs that are not guaranteed in opportunistic encounters with real patients.

Positive evaluations were received for attractiveness, efficiency, dependability, stimulation, and novelty. Efficiency, dependability, stimulation, and perspicuity are parameters that are relevant for learning, and positive evaluations were received for the first 3. However, neutral evaluations were received for perspicuity (quality of being clear and easy to understand). It is postulated that many students had difficulty with the pinching gesture required to select specific options when operating the HoloLens. Despite demonstrating the technique to the students a few times, a few of them were unable to grasp it. This was deemed to be a technical limitation of the device and is expected to improve in subsequent updates. Very positive evaluations were received for novelty, which is not surprising in view of the fact that students are not exposed to MR in their usual clinical rotations. When comparing the UEQ results of the 2 groups, there was no statistically significant difference in the evaluation. As such, we can conclude that there was no translatable interaction between the study design and perceptions of the software.

### Implications of the Findings

#### Deliberate Practice and Experiential Learning

EyelearnMR, being a type of simulation learning, facilitates deliberate practice in a controlled environment and allows for learning through problem-solving [23]. A systematic review by Cook et al [20] found that simulation training in health profession education was consistently associated with large positive effects on outcomes of knowledge, skills, and behaviors and moderate positive effects for patient-related outcomes, cementing its role in health care education.

In the EyelearnMR group, the learning module was presented to the students in a structured and guided fashion in accordance with pedagogical best practices, and they were allowed to practice their examinations at their own pace, repeating specific cases if necessary. Conversely, the control group only had access to traditional clinical skill tutorials and clinical sessions, which typically use either simulated or real patients. These patients may not allow for enough deliberate practice time due to fatigue or time constraints. In a clinical tutorial group setting, the teacher-to-student ratio is typically 1:7, which limits the number of attempts that students may be able to practice [24].

#### Reproducibility of Clinical Signs With a Comprehensive Selection of Conditions

Making a diagnosis from physical examination involves applying a set of clinical examination skills consistently, then evaluating the findings and reaching a conclusion. Repeated deliberate practice of the examination steps improves consistency, and exposure to a variety of conditions builds a knowledge base for appropriate diagnosis.

In the control group (and traditional clinical teaching environments), the range of conditions that a student may encounter is unequal across the student population. Simulated patients (often volunteers) are not able to fully replicate critical, life-threatening clinical signs for effective skill transfer, especially for a topic such as ocular examination. The alternative is to have students examine real patients with the actual condition. While this is done opportunistically in the clinic for all students, it is impossible for them to be exposed to the same conditions with the same degree of significant findings. However, in the EyelearnMR group, every student was given the opportunity to examine a defined and comprehensive set of conditions. They were able to fully experience and learn from “patients” displaying consistent and reproducible clinical signs [25]. This facilitated a broad knowledge base on which to build their diagnostic competencies [26]. This is in contrast with the control group, which had to rely on chance encounters with such patients in the clinical setting, likely with varying physical findings.

The ability of the EyelearnMR module to reproduce the same clinical signs allows for truly experiential learning and deliberate practice for the students, increasing the effectiveness of the teaching and knowledge retention. This forms a strong foundation for the understanding of the underlying concepts, which translated to better scores on the final assessment.

### Comparison to the Literature

There are existing virtual and augmented reality applications in ophthalmology, but these are mainly on surgical simulation such as cataract surgery training or anatomical visualization [27]. However, EyelearnMR targets the ocular motility clinical examination—a skill set often neglected in current platforms [28]. It enables users to interact with life-sized, holographic patients with varied and reproducible motility disorders. This provides students with the opportunity to practice their examination techniques repeatedly in a realistic and reproducible environment and may help build their confidence and develop diagnostic skills in a more consistent and reliable way.

### Strengths and Limitations

This is a novel study that investigates the use of MR to teach ocular motility testing. To our knowledge, no existing simulator can perform this task. A scoping review by Krutsinger et al [29] on virtual reality–based medical education in ophthalmology summarized its use mainly for surgical training; clinical applications in diagnosis; counseling; and physical examination, including simulated pupil examination, simulation-based slit lamp training, and ophthalmoscopy [23]. As such, this study addresses an important gap by examining the use of MR to teach ocular motility testing, an area not covered by current simulators or virtual reality platforms. By combining real and virtual environments, MR enables learners to appreciate and interact with extraocular movements in 3 dimensions, supporting a deeper understanding of spatial relationships and examination techniques that are difficult to convey through traditional or screen-based simulations.

The technical limitations of the HoloLens 2 headset include limited field of view and low battery life. The device has a field of view of 43° horizontally compared to the human’s field of view of 135°. Therefore, users must adjust their head angles to ensure that the targets are within the device’s field of view. On average, battery life can only be sustained for 2 hours of active use. Inclusive of setup time, a device would usually be near the end of its battery life at the end of the assessment. Student feedback highlighted issues with tracking accuracy despite the device’s capabilities for both hand and eye tracking. Cost is also a limitation as each HoloLens 2 headset can be costly and adequate funding is required to support such deployment. Finally, the EyelearnMR evaluated a single examination skill (ocular motility) with tightly defined parameters. This was done purposefully to maintain the scientific rigor in the study and reduce other potential confounders. Therefore, the results of this study cannot be extrapolated to other forms of clinical skill examination at this point in time. Future studies will be useful for exploring broader applications of MR in medical education.

MR can also create a potentially dangerous environment if users are not careful; for example, users may not be aware of their surroundings and could trip, fall, or walk into objects. This is more of a concern in virtual reality, where the user is unable to see the actual surroundings, whereas in MR, the user can observe their real environment.

Our cohort consisted of fourth-year medical students from a single medical school rotation, and participation was opt-in. This introduces 2 potential issues. First, the sample size was modest, and group allocation by clinical batch may mean that unmeasured differences between groups (eg, baseline motivation, prior exposure to neuro-ophthalmology, or varying tutorial quality) could influence outcomes. Second, because participation was voluntary, there may have been self-selection bias; students who agreed to participate could differ systematically from those who declined, particularly in interest or confidence in ophthalmology.

In terms of study design, as this study was on an opt-in basis for the students who rotated to the hospital, the recruited sample size was not balanced between the 2 groups: 24 in the control arm and 30 in the EyelearnMR arm. This imbalance in sample size could cause selection bias and potentially reduce statistical power and precision. Although the completed questionnaires were retrieved from the students, a limitation of this study was that it assumed academic integrity, trusting that students would refrain from sharing the scenarios or questions with future student groups rotating through the ophthalmology posting.

The assessment was administered at different time points for the 2 groups (days 8 and 9 vs days 11 and 12). Although this reflected how EyelearnMR would realistically be integrated into the rotation, we cannot exclude a potential recency effect. However, the assessment targeted application of examination technique and diagnostic reasoning rather than simple recall, and the control group had continuous clinical exposure and tutorials up to the end of the posting.

Although video-based assessments standardize stimuli, they may not fully represent the variability observed in real patients. Subtle motility deficits may appear different when captured on video rather than during a face-to-face examination. Additionally, the bespoke nature of the assessment means that it has not yet undergone formal psychometric validation (eg, reliability testing and item difficulty calibration). This may affect reproducibility if used in different settings or with different cohorts.

It is also important to acknowledge that the observed improvement cannot be attributed solely to the MR software itself. EyelearnMR was delivered during a structured teaching session that incorporated pedagogical best practices, including guided demonstration, self-paced exploration, and opportunities for repeated practice with immediate visual feedback. Any of these components may have contributed to the improved performance. This study was not designed to isolate the individual effects of software features, instructional approach, or practice opportunities, and therefore, the greatest effect on learning cannot be determined from our data alone.

### Conclusions

On the basis of this study, EyelearnMR with traditional teaching is noninferior to traditional teaching alone. It provided a comparable experience and equally supported learning objectives. It is an effective supplementary teaching tool in ophthalmic education and may confer additional learning benefits to those of a traditional clinical posting, especially in the field of clinical examination technique.

## Supplementary material

10.2196/71338 Multimedia Appendix 1 Video assessment questions and the user experience questionnaire.

## Abbreviations

MR: mixed reality
UEQ: User Experience Questionnaire
