# Supplementary material for: Application of Mixed Reality for Ophthalmic Clinical Skills and Diagnosis: Prospective Study
Source: JMIR Med Educ. 2026 Mar 3;12:e71338. doi: 10.2196/71338 (PMC12978906; doi:10.2196/71338)

**Supplementary**

Assessment

**Question 1**

1. What is wrong with this technique? (Technique)

A. Did not lift up eyelids on up gaze

B. Did not lift up eyelids on down gaze

C. Did not lift up eyelids on left gaze

D. Did not lift up eyelids on right gaze

2. Which gaze was not tested? (Technique)

A. Down and right gaze

B. Up and right gaze

C. Up and left gaze

D. Down and left gaze

3. Which other part of the exam was not performed? (Technique)

A. Divergence

B. Convergence

C. Right gaze

D. Left gaze

**Question 2**

1. What do you see on general inspection? (Examination Findings)

A. Right partial ptosis

B. Left partial ptosis

C. Right esotropia

D. Left esotropia

2. What ocular motility limitation is/are there? (Examination Findings)

i) Left adduction deficit

ii) Right adduction deficit

iii) Left abduction deficit

iv) Left depression deficit

v) Left elevation deficit

A. ii

B. ii and iii

C. i, ii and v

D. i, iv and v

3. What did you notice about the eyelid positions while testing the horizontal gazes? (Examination Findings)

A. Left upper eyelid became higher on adduction

B. Left upper eyelid became lower on adduction

C. Right upper eyelid became higher on abduction

D. Right upper eyelid became lower on adduction

4. What is the most likely diagnosis? (Pathology)

A. Cranial nerve 6 palsy

B. Cranial nerve 2 palsy

C. Cranial nerve 3 palsy

D. Internuclear ophthalmoplegia

**Question 3**

1. What ocular motility limitation is there? (Examination Findings)

A. Right adduction deficit

B. Right abduction deficit

C. Left adduction deficit

D. Left abduction deficit

2. Which extraocular muscle is affected? (Pathology)

A. Left superior oblique

B. Right superior oblique

C. Left lateral rectus

D. Right lateral rectus

3. What is the most likely diagnosis? (Pathology)

A. Cranial nerve 3 palsy

B. Cranial nerve 4 palsy

C. Cranial nerve 6 palsy

D. Cranial nerve 7 palsy

**Question 4**

1. What do you see on general inspection? (Examination Findings)

i) Left upper lid ectropion

ii) Right upper lid retraction

iii) Right conjunctival injection

iv) Left conjunctival injection

A. i and ii

B. ii and iii

C. i and iv

D. i, ii and iii

2. What ocular motility limitation is there? (Examination Findings)

A. Right abduction deficit

B. Right adduction deficit

C. Left abduction deficit

D. Left elevation deficit

3. What is the sign elicited when the patient looks down? (Examination Findings)

A. Inferior scleral show

B. Lid lag

C. Temporal flare

D. Hypotropia

4. What is the most likely diagnosis? (Pathology)

A. Thyroid eye disease

B. Internuclear ophthalmoplegia

C. Cranial nerve 3 palsy

D. Cranial nerve 4 palsy

**Question 5**

1. What ocular motility limitation is there? (Examination Findings)

A. Right adduction deficit

B. Left adduction deficit

C. Right abduction deficit

D. Left abduction deficit

2. What other sign is present in left gaze? (Examination Findings)

A. Right eye adduction nystagmus

B. Left eye abduction nystagmus

C. Right eye intorsion

D. Left eye intorsion

3. What is the most likely diagnosis? (Pathology)

A. Cranial nerve 2 palsy

B. Cranial nerve 3 palsy

C. Cranial nerve 6 palsy

D. Internuclear ophthalmoplegia

User Experience Questionnaire


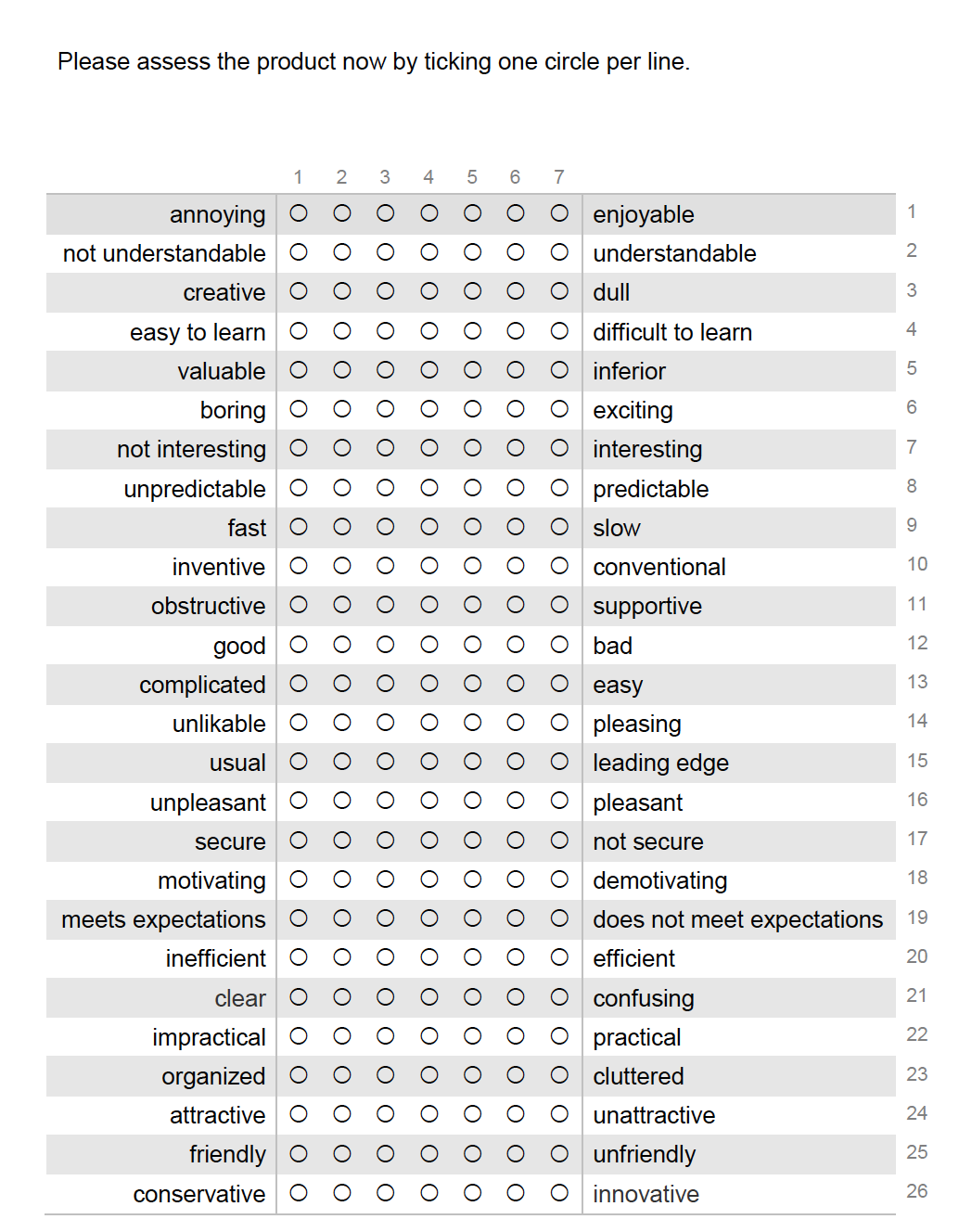

Supplement: Multimedia Appendix 1 [file mededu-v12-e71338-s001.docx]
